# Steroid-Responsive Encephalopathy Associated With Autoimmune Thyroiditis Presenting As Acute Psychosis: A Diagnostic Pitfall

**DOI:** 10.7759/cureus.109061

**Published:** 2026-05-17

**Authors:** Ismael Palacio, Oren G Nedjar, Natalia C Flores Soto, Astrid Iglesias, Sebastian Campuzano Arias, Marvi A Surksha, Miguel Barros, Angelica Ludena, Eugenio Angueira-Serrano, George J Michel

**Affiliations:** 1 Internal Medicine, Larkin Community Hospital, South Miami, USA; 2 Internal Medicine, Nova Southeastern University Dr. Kiran C. Patel College of Osteopathic Medicine (NSU KPCOM), Davie, USA; 3 Internal Medicine, University of Medicine and Health Sciences (UMHS), Miami, USA; 4 Endocrinology, Larkin Community Hospital Palm Springs Campus, Hialeah, USA

**Keywords:** autoimmune encephalopathy, corticosteroid therapy, hashimoto encephalopathy, hashimoto thyroiditis, sreat, steroid-responsive encephalopathy associated with autoimmune thyroiditis, thyroid antibodies

## Abstract

Steroid-responsive encephalopathy associated with autoimmune thyroiditis (SREAT) is a rare autoimmune encephalopathy characterized by heterogeneous neuropsychiatric manifestations. We describe the case of a 59-year-old woman with a history of anxiety and severe alcohol use disorder who presented with acute psychosis, marked behavioral disorganization, and cognitive impairment initially attributed to alcohol withdrawal, metabolic disturbance, or primary psychiatric illness. Despite extensive evaluation, including negative infectious, toxicologic, metabolic, structural, and paraneoplastic workups, no clear etiology was identified. Additional findings, including a reactive HIV screening assay and elevated vitamin B12 levels, further complicated the diagnostic process. Thyroid studies revealed markedly elevated anti-thyroid peroxidase and anti-thyroglobulin antibodies with subclinical hypothyroidism, raising suspicion for SREAT. Following initiation of high-dose intravenous (IV) corticosteroids, the patient demonstrated rapid and marked improvement in mental status within 48 hours, supporting an immune-mediated process. Subsequent cerebrospinal fluid analysis, electroencephalography, and brain imaging remained unremarkable. This case highlights the diagnostic challenges of SREAT, particularly in patients with comorbid psychiatric and substance use disorders, and underscores the importance of considering autoimmune encephalopathy in cases of unexplained neuropsychiatric decline. Early recognition and timely immunosuppressive therapy may lead to significant clinical recovery and prevent prolonged morbidity.

## Introduction

Autoimmune thyroid disease (AITD) comprises a group of disorders characterized by immune-mediated dysfunction of the thyroid gland [1]. The two principal forms are Hashimoto thyroiditis and Graves disease [1]. Hashimoto thyroiditis, the most common cause of autoimmune hypothyroidism, results from lymphocytic destruction of thyroid tissue, whereas Graves disease produces hyperthyroidism through stimulating antibodies directed against the thyroid-stimulating hormone receptor [1,2]. Hashimoto thyroiditis is typically diagnosed by the presence of circulating thyroid autoantibodies, most commonly anti-thyroid peroxidase (anti-TPO) and anti-thyroglobulin antibodies, often in combination with thyroid function abnormalities and, in some cases, supportive ultrasonographic findings [3]. Steroid-responsive encephalopathy associated with autoimmune thyroiditis (SREAT), also referred to as Hashimoto’s encephalopathy, is an uncommon neurological disorder associated with autoimmune thyroid disease. Hashimoto thyroiditis is the most common cause of hypothyroidism in iodine-sufficient regions and results from antibody-mediated destruction of the thyroid gland, often involving anti-thyroid peroxidase and anti-thyroglobulin antibodies. The diagnosis of Hashimoto thyroiditis is typically based on clinical features of hypothyroidism in conjunction with elevated thyroid-stimulating hormone levels, reduced free thyroxine concentrations, and the presence of thyroid autoantibodies [4-6]. Although the disease primarily affects the endocrine system, it has also been associated with neurological manifestations, including SREAT [7-9]. SREAT is characterized by a wide spectrum of neuropsychiatric manifestations, including cognitive impairment, seizures, behavioral disturbances, and psychosis [10-12]. Although classified among autoimmune encephalitides, the underlying pathophysiological mechanisms remain incompletely understood and are believed to reflect immune-mediated central nervous system dysfunction rather than direct thyroid hormone effects [10-12]. In fact, although thyroid antibodies are strongly associated with SREAT, they are generally considered markers of immune dysregulation rather than directly pathogenic mediators [13].

The estimated prevalence of SREAT is approximately 2.1 per 100,000 individuals [14]. The disorder shows a marked female predominance and has been reported across a broad age range, from adolescence to older adulthood, with a mean age of onset between 40 and 55 years and a female-to-male ratio of approximately 5:1 [15-17]. SREAT is frequently associated with other autoimmune conditions, including systemic lupus erythematosus, type 1 diabetes mellitus, and Sjögren’s syndrome [4].

Clinical manifestations are diverse and often nonspecific, contributing to frequent misdiagnosis or delayed recognition [7,11,17]. Presentations commonly include psychiatric symptoms such as hallucinations, paranoia, and behavioral disorganization, as well as neurological manifestations including altered levels of consciousness, seizures, cognitive decline, movement disorders, cerebellar ataxia, speech impairment, and autonomic dysfunction [7,18]. Because of this heterogeneity, several classification systems have been proposed. The traditional two-type model distinguishes a vasculitic or stroke-like form from a diffuse progressive form characterized by psychiatric predominance [18]. More recently, a four-syndrome model has categorized clinical presentations into psychiatric, encephalopathic, new-onset refractory status epilepticus, and limbic encephalitis phenotypes [8].

Despite its association with autoimmune thyroid disease, SREAT is independent of thyroid functional status and may occur in euthyroid, hypothyroid, or hyperthyroid states. Elevated anti-thyroid antibodies, particularly anti-thyroid peroxidase antibodies, are considered markers of immune dysregulation rather than direct mediators of central nervous system injury. Despite extensive evaluation, ancillary studies remained unrevealing in our patient, consistent with the known diagnostic limitations of conventional testing in SREAT [6].

Diagnostic criteria typically include subacute encephalopathy, elevated anti-thyroid antibodies, absence of neuronal antibodies, and exclusion of alternative causes. Although responsiveness to corticosteroid therapy is commonly observed, it is not universal and should not be regarded as a defining diagnostic feature [8].

Delayed recognition of SREAT may lead to prolonged morbidity, unnecessary psychiatric treatment, and extensive diagnostic testing, whereas early identification and immunotherapy, most often corticosteroids, can result in rapid clinical improvement and prevention of irreversible cognitive decline [7]. Because neuroimaging, cerebrospinal fluid studies, and electroencephalography findings are frequently nonspecific, clinicians must maintain a high index of suspicion, particularly in patients presenting with treatment-refractory neuropsychiatric symptoms and coexisting autoimmune markers [8,9].

Here, we present a diagnostically challenging case of SREAT in which prominent psychiatric manifestations, metabolic abnormalities, alcohol use disorder, and misleading laboratory findings initially obscured the underlying autoimmune etiology, underscoring the importance of maintaining a broad differential diagnosis and performing recurrent diagnostic reassessment in treatment-refractory neuropsychiatric syndromes.

## Case presentation

A 59-year-old woman with a 2-year history of anxiety and a 6-month severe alcohol use disorder, reportedly consuming approximately two bottles of whiskey daily, presented to the Emergency Department with an acute onset of bizarre behavior, paranoia, marked disorganization, and apparent psychosis. She was found naked, seated on the floor of her room, covered in feces that she had smeared onto her arms, and was observed speaking to herself. Information collected by collateral denied any history of head trauma, prior psychiatric or neurological hospitalizations, or use of substances other than alcohol. Her anxiety disorder had been self-treated with alprazolam 0.5 mg taken as needed, typically once daily, for approximately two years, without formal psychiatric supervision. Regarding alcohol use disorder, the patient had not previously undergone pharmacologic treatment, rehabilitation programs, or structured addiction therapy prior to this hospitalization.

On arrival, the patient appeared disheveled and was oriented only to self, demonstrating a blunted affect and marked behavioral disorganization. Psychiatric evaluation was limited due to impaired attention and severely disorganized thought processes. Speech was sparse, tangential, and frequently trailed off mid-sentence. Her responses were inconsistent and incoherent, preventing reliable history-taking. She intermittently lay in bed with her eyes closed, mumbling incomprehensibly.

A comprehensive review of systems could not be reliably obtained due to the patient’s altered mental status. Vital signs on presentation included blood pressure 128/66 mm Hg (supine), heart rate 101 beats per minute, respiratory rate 17 breaths per minute, temperature 98.6 °F, and oxygen saturation of 98% on room air. Physical examination revealed a fine perioral tremor, intermittent head tremor, and eyelid fluttering with eyes closed. Examination of the neck revealed no palpable thyroid enlargement, nodules, or tenderness, and the thyroid gland appeared normal in size, consistent with the unremarkable thyroid ultrasound findings.

Mental status examination demonstrated impaired attention, disorganized thought processes, and an inability to participate in formal cognitive testing. Thought process and content, including assessment of suicidality or homicidality, could not be reliably evaluated. Insight and judgment were similarly unassessable. Although she intermittently denied hallucinations or paranoia, the reliability of her responses was poor, and she was unable to participate meaningfully in a comprehensive psychiatric assessment. Formal psychometric testing was not performed during the acute phase because the patient was unable to participate reliably due to severe cognitive and behavioral disorganization. 

Collateral information obtained from the patient’s brother indicated that he had spoken with her by phone five days prior to presentation, at which time she was reportedly oriented, with coherent speech and appropriate affect. The patient resides with a roommate. She maintained regular communication with her brother, who served as a key collateral historian during the initial evaluation. She reported no current romantic relationship and denied any history of legal problems or illicit drug use, with alcohol being the only substance used. Prior to hospitalization, the patient was independent in her activities of daily living and was employed as a logistics coordinator. Family medical history was noncontributory, with no known history of thyroid disease, autoimmune disorders, neurological illness, or major psychiatric conditions among first-degree relatives.

**Figure 1:**
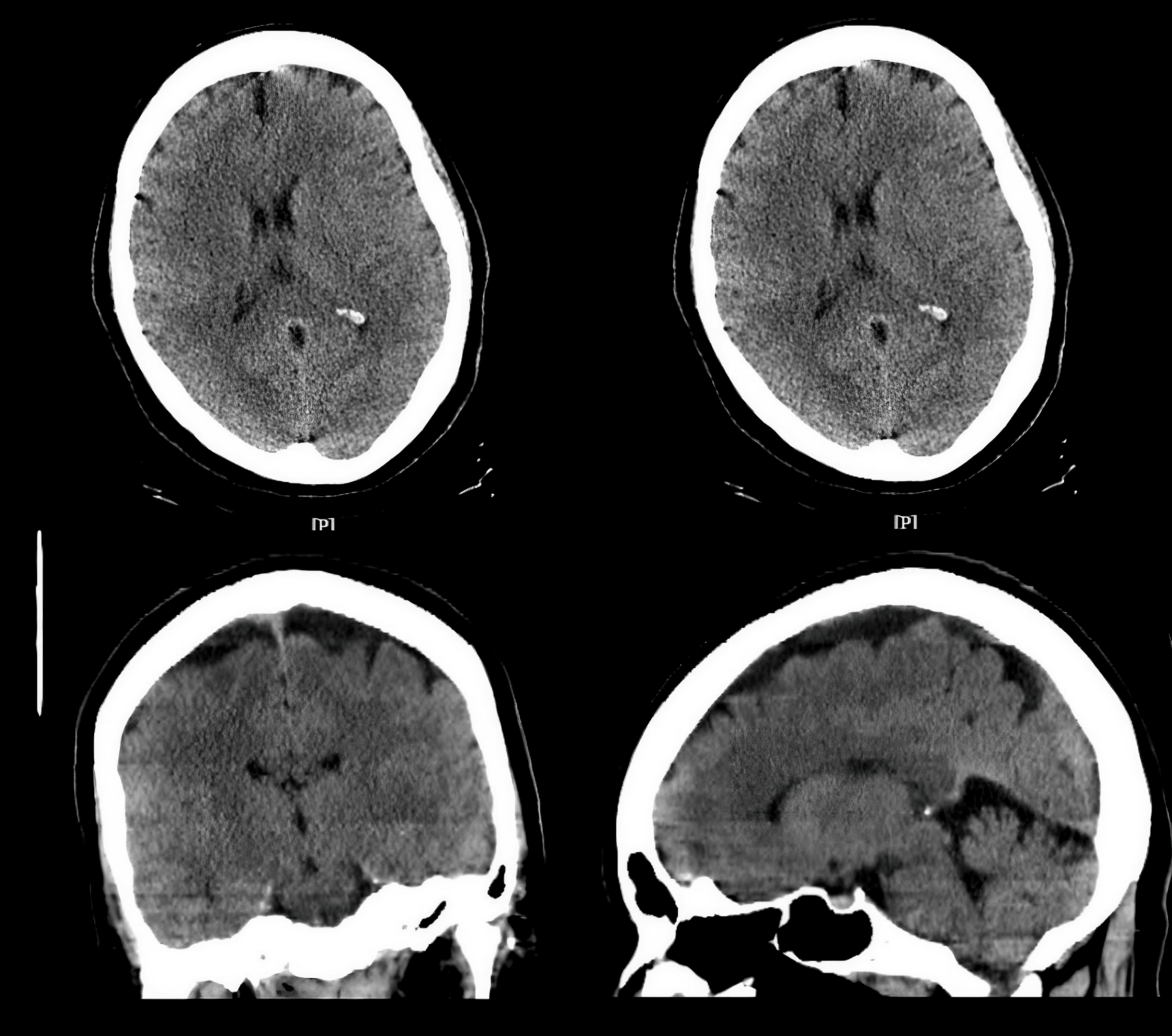
Non-contrast computed tomography (CT) of the brain. There was no evidence of acute intracranial hemorrhage, mass effect, or midline shift. Ventricular size and cortical sulci appear within normal limits for the patient's age, and no focal parenchymal abnormalities were identified.

Initial diagnostic workup included a non-contrast CT scan of the head, which showed no evidence of intracranial hemorrhage, mass effect, or midline shift (Figure 1). The metabolic panel did not show significant electrolyte, hepatic, or renal abnormalities (Table 1). A mild elevation in creatine kinase was noted and attributed to psychomotor agitation (Table 1). On presentation, she was hypoglycemic, likely secondary to starvation physiology, accompanied by mild ketosis and metabolic acidosis, which was resolved with intravenous (IV) isotonic fluids (0.9% normal saline)(Table 1 and Table 2). Urinalysis, urine toxicology screening, and pregnancy testing were negative (Table 2). While routine toxicology screening was negative, exposure to substances not captured on standard panels could not be definitively excluded.

**Table 1:** Metabolic, hepatic, and hematologic profiles (F) = female reference range

Category	Test	Result	Reference Range
Electrolytes	Sodium (Na)	139 mEq/L	136–145 mEq/L
	Potassium (K)	3.6 mEq/L	3.5–5.0 mEq/L
	Chloride (Cl⁻)	105 mEq/L	98–107 mEq/L
	Calcium (Ca)	9.7 mg/dL	8.5–10.5 mg/dL
	Magnesium (Mg)	2.1 mg/dL	1.7–2.2 mg/dL
	Phosphorus (Phos)	3.5 mg/dL	2.5–4.5 mg/dL
	Bicarbonate (HCO₃)	18 mEq/L	22–29 mEq/L
Renal/Metabolic	Blood urea nitrogen (BUN)	22 mg/dL	7–20 mg/dL
	Creatinine	0.78 mg/dL	0.5–1.1 mg/dL
	Glomerular filtraton rate (GFR)	80 mL/min/1.73m²	≥60 mL/min/1.73m²
	Osmolarity	282 mOsm/kg	275–295 mOsm/kg
	BUN/Creatinine (Cr) Ratio	33	10–20
	Glucose	65 mg/dL	70–100 mg/dL
	Creatine kinase (CK)	322 U/L	30–150 U/L (F)
Liver/Proteins	Aspartate aminotransferase (AST)	33 U/L	10–40 U/L
	Alanine aminotransferase (ALT)	43 U/L	7–40 U/L
	Alkaline phosphatase	87 U/L	44–147 U/L
	Total bilirubin	0.9 mg/dL	0.2–1.2 mg/dL
	Total protein	7.4 g/dL	6.3–8.2 g/dL
	Albumin	4.4 g/dL	3.5–5.0 g/dL
	Globulin	3.0 g/dL	2.0–3.5 g/dL
	Albumin/globulin (A/G) Ratio	1.5	1.2–2.2
	Ammonia	<9 µmol/L	15–45 µmol/L
Lipids	Total Cholesterol	237 mg/dL	<200 mg/dL
	Triglycerides	54 mg/dL	<150 mg/dL
	High-density lipoprotein (HDL)	61 mg/dL	≥60 mg/dL (optimal, F)
	Low-density lipoprotein (LDL)	165 mg/dL	<100 mg/dL (optimal)
	Very low-density lipoprotein (VLDL)	10.8 mg/dL	2–30 mg/dL
CBC (Hematology)	WBC	10.01 × 10³/µL	4.5–11.0 × 10³/µL
	RBC	4.41 × 10⁶/µL	3.9–5.0 × 10⁶/µL (F)
	Hemoglobin (HGB)	12.6 g/dL	12.0–16.0 g/dL (F)
	Hematocrit (HCT)	38.5%	36–46% (F)
	Mean corpuscular volume (MCV)	87.3 fL	80–100 fL
	Mean corpuscular hemoglobin (MCH)	28.6 pg	27–33 pg
	Mean corpuscular hemoglobin concentration (MCHC)	32.7 g/dL	32–36 g/dL
	Red cell distribution width (RDW)	13.4%	11.5–14.5%
	Red Cell Distribution Width Standard Deviation (RDWSD)	43.8 fL	37–54 fL
	Platelets (PLT)	292 × 10³/µL	150–400 × 10³/µL
	Mean platelet volume (MPV)	11.5 fL	7.5–12.5 fL
CBC Differential	Neutrophils (NEUT)	63.8%	50–70%
	Lymphocytes (LYMPH)	25%	20–40%
	Monocytes (MONO)	6.2%	2–10%
	Eosinophils (EOS)	3.4%	1–4%
	Basophils (BASO)	0.6%	0–1%
	Neutrophils absolute count (NEUT abs)	6.39 × 10³/µL	1.8–7.7 × 10³/µL
	Lymphocytes absolute count (LYMPH abs)	2.5 × 10³/µL	1.0–4.8 × 10³/µL
	Monocytes absolute count (MONO abs)	0.62 × 10³/µL	0.2–0.95 × 10³/µL
	Eosinophils absolute count (EOS abs)	0.34 × 10³/µL	0.05–0.5 × 10³/µL
	Basophils absolute count (BASO abs)	0.06 × 10³/µL	0–0.1 × 10³/µL
Inflammatory/Other	Erythrocyte sedimentation rate (ESR)	17 mm/hr	0–20 mm/hr (F)
	C-reactive protein (CRP)	<0.5 mg/dL	<1.0 mg/dL
	Lactate dehydrogenase (LDH)	211 U/L	140–280 U/L
	Prothrombin time (PT)	11.2 sec	11.0–13.5 sec
	International normalized ratio (INR)	1.0	0.8–1.1

**Table 2:** Screening, toxicology, and urinalysis

Category	Test	Result
Pregnancy	Urine chorionic gonadotropin (UCG)	Negative
Toxicology	Amphetamines	Negative
	Barbiturates	Negative
	Benzodiazepines	Negative
	Cocaine	Negative
	Methadone	Negative
	Natural opiates	Negative
	Phencyclidine	Negative
	Tetrahydrocannabinol	Negative
	Ethanol	<1 mg/dL
Urinalysis	Appearance	Hazy
	Specific gravity	>1.030
	Ketones	3+
	Protein	Trace
	Infection screen	Negative
	WBCs	<5/high-power field
	RBCs	Present
	Granular casts	None
	Bacteria	None
	Squamous epithelial cells	Many

The timing of the patient’s last alcohol consumption prior to presentation was unknown. However, alcohol levels were < 1 mg/dL. Vitamin D was low at 24.8 ng/mL, consistent with insufficiency, and supplementation was initiated (Table 3). Blood and urine cultures were obtained, and empiric antimicrobial therapy with cefepime 2 g administered IV every 8 hours was initiated due to concern for possible sepsis in the setting of acute altered mental status and unclear infectious etiology, which was subsequently discontinued at 48 hours after negative culture results were received. Supportive management also included IV isotonic fluids (0.9% normal saline) administered at approximately 75-100 mL/hour during the initial hospitalization period for volume support and metabolic stabilization.

**Table 3:** Nutritional and Vitamin Assessment

Test	Result	Reference Range
Vitamin B12	993 pg/mL	200–900 pg/mL
Folate	>20.0 ng/mL	>4.0 ng/mL
Vitamin B1 (Thiamine)	98 nmol/L	70–180 nmol/L
Vitamin D (25-OH)	24.8 ng/mL	30–100 ng/mL

Vitamin B12 levels were elevated at 993 pg/mL on initial laboratory testing prior to administration of vitamin supplementation or other medications (Table 3). Although nonspecific, this degree of elevation has been associated with malignancy, hepatocellular disease, or myeloproliferative processes [19]. Given an unexplained vitamin B12 elevation, additional imaging was obtained to evaluate for occult hepatic, renal, or hematologic pathology. Computed tomography of the abdomen and pelvis revealed no acute abnormalities, and complementary abdominal and renal ultrasounds were also unremarkable.

Given the patient’s history of heavy alcohol use and acute neuropsychiatric changes, the initial differential diagnosis included Wernicke encephalopathy, delirium tremens, metabolic encephalopathy, and primary psychosis. Empiric treatment for suspected alcohol-related encephalopathy was initiated with IV thiamine at a dose of 500 mg every 8 hours for three days, followed by 250 mg daily, and subsequently transitioned to 100 mg orally once daily. Benzodiazepine therapy consisted of lorazepam 2 mg administered intramuscularly every 4 hours as needed for agitation or seizure prophylaxis, in addition to scheduled lorazepam 2 mg orally every 8 hours during hospitalization. The patient was monitored using the Clinical Institute Withdrawal Assessment for Alcohol (CIWA) protocol every 6 hours. CIWA scores during hospitalization remained below 8, indicating mild or absent alcohol withdrawal symptoms. CIWA scores were interpreted as follows: scores <8 indicate minimal withdrawal, 8-15 moderate withdrawal, and >15 severe withdrawal requiring aggressive management [20].

Serial neurologic evaluations were performed throughout hospitalization. On initial neurologic examination, the patient was alert but disoriented (oriented only to person), with impaired attention, disorganized speech, and difficulty following commands. Cranial nerve examination was intact, and motor strength was preserved bilaterally with normal muscle bulk and tone. Coordination assessment was limited due to impaired cooperation. Neurologic status was monitored with neurologic checks every 6 hours, which initially demonstrated persistent confusion and impaired concentration. Following the initial evaluation in the emergency department, the patient was admitted to the inpatient medical service for further diagnostic evaluation and management, with involvement from neurology, psychiatry, and endocrinology teams.

Psychiatry consultation led to initiation of divalproex sodium 250 mg every eight hours, haloperidol 5 mg twice daily, and quetiapine 50 mg at bedtime for suspected acute psychosis. However, the following day, the patient exhibited worsening restlessness, disorientation, and agitation despite pharmacologic management, requiring intermittent intramuscular lorazepam 2 mg administration.

A comprehensive infectious disease evaluation was conducted. Serologic testing for syphilis, hepatitis A, hepatitis B, and hepatitis C was nonreactive. However, HIV antigen/antibody screening returned reactive, prompting confirmatory HIV-1 PCR testing. Additional viral encephalitis screening, including herpes simplex virus 1 and 2 polymerase chain reaction (HSV-1/2 PCR), cytomegalovirus polymerase chain reaction (CMV PCR), Epstein-Barr virus immunoglobulin M (EBV IgM), varicella-zoster virus immunoglobulin M (VZV IgM), and COVID-19 testing, was negative. Antinuclear antibody testing was also nonreactive (Table 4).

**Table 4:** Infectious disease and immunology workup HIV Ag/Ab: Human immunodeficiency virus antigen/antibody; HIV-1 RNA PCR: Human immunodeficiency virus type 1 ribonucleic acid polymerase chain reaction; RPR: Rapid plasma reagin; HAV IgM: Hepatitis A virus immunoglobulin M; HBsAg: Hepatitis B surface antigen; HBc IgM: Hepatitis B core immunoglobulin M; HCV Ab: Hepatitis C virus antibody; HSV-1/2 PCR: Herpes simplex virus 1 and 2 polymerase chain reaction; CMV PCR: Cytomegalovirus polymerase chain reaction; EBV IgM: Epstein-Barr virus immunoglobulin M; VZV IgM: Varicella-zoster virus immunoglobulin M; COVID-19: Coronavirus disease 2019; ANA: Antinuclear antibody; CD4: Cluster of differentiation 4; CD8: Cluster of differentiation 8

Category	Test	Result	Reference Range
HIV Screening	HIV Ag/Ab (Initial)	Reactive	Nonreactive
	HIV-1 RNA PCR	<20 copies/mL	Undetected
	HIV Ag/Ab (Repeat)	Negative	Negative
Syphilis	Treponema Antibody	Nonreactive	Nonreactive
	RPR	Nonreactive	Nonreactive
Hepatitis	HAV IgM	Negative	Negative
	HBsAg	Negative	Negative
	HBc IgM	Negative	Negative
	HCV Ab	Negative	Negative
Viral/Other	HSV-1/2 PCR	Negative	Negative
	CMV PCR	Negative	Negative
	EBV IgM	Negative	Negative
	VZV IgM	Negative	Negative
	COVID-19	Negative	Negative
	Toxoplasma	Negative	Negative
	Cryptococcus Ag	Negative	Negative
	JC Virus	Negative	Negative
Immunology	ANA	Nonreactive	Nonreactive
	CD4 Count	635 cells/µL	500–1,500 cells/µL
	CD8 Count	715 cells/µL	150–1,000 cells/µL
	CD4/CD8 Ratio	0.88	1.0–2.5

Thyroid evaluation revealed an elevated thyroid-stimulating hormone (TSH) level of 7.20 µIU/mL with a normal free thyroxine (T4) level (1.36 ng/dl), consistent with subclinical hypothyroidism. The thyroid ultrasound was unremarkable. Given her neuropsychiatric presentation, autoimmune thyroid disease was considered. Reflex antibody testing demonstrated markedly elevated thyroid peroxidase (TPO) and thyroglobulin antibodies (TgAb) (>600 IU/mL) with negative thyroid-stimulating immunoglobulin (TSI <0.10), and TSH receptor antibodies (TRAb <1.10), raising suspicion for steroid-responsive encephalopathy associated with autoimmune thyroiditis (SREAT) (Table 5).

**Table 5:** Endocrine and pituitary function

Test	Result	Reference Range
Thyroid-stimulating hormone (TSH) (Initial)	7.2 µIU/mL	0.4–4.0 µIU/mL
Free thyroxine — FT4 (Initial)	1.36 ng/dL	0.8–1.8 ng/dL
TSH (Repeat)	7.08 µIU/mL	0.4–4.0 µIU/mL
Free T4 — FT4 (Repeat)	2.33 ng/dL	0.8–1.8 ng/dL
Free triiodothyronine — FT3	7.19 pg/mL	2.3–4.2 pg/mL
Thyroid peroxidase (TPO) antibody	>600 IU/mL	<35 IU/mL
Thyroglobulin antibody (TgAb)	>600 IU/mL	<20 IU/mL
Thyroid-stimulating immunoglobulin (TSI)	<0.10	<0.55
Thyroid-stimulating hormone receptor antibody (TRAb)	<1.10 IU/L	<1.75 IU/L
Cortisol (AM (morning))	16.7 µg/dL	6–23 µg/dL (AM)
Prolactin (PRL)	25 ng/mL	2–29 ng/mL (F)
Follicle-stimulating hormone (FSH)	88.5 IU/L	25.8–134.8 IU/L (postmenopausal)
Luteinizing hormone (LH)	48.6 IU/L	7.7–58.5 IU/L (postmenopausal)
Insulin-like growth factor 1 (IGF-1)	42 ng/mL	~52–228 ng/mL (age 55–60)*
C-Peptide	3.2 ng/mL	0.8–3.5 ng/mL
Insulin	11.5 µIU/mL	2.6–24.9 µIU/mL
Hemoglobin A1c	5.3%	<5.7%

Given concern for HIV encephalopathy versus autoimmune thyroiditis-associated encephalopathy, high-dose intravenous corticosteroid therapy was initiated. Because of the patient’s rapidly progressive symptoms and lack of response to initial management, corticosteroids were initiated empirically while other etiologies were being excluded. The patient received IV methylprednisolone infusion at a dose of 1 g daily for five consecutive days. Following completion, oral prednisone was initiated at 40 mg once daily. The dose was subsequently tapered gradually based on clinical response, with reductions of 10 mg per week at higher doses, followed by 2.5 mg per week once the dose reached 10 mg daily, and 1 mg per week once the dose reached 5 mg daily, while monitoring morning cortisol levels to assess hypothalamic-pituitary-adrenal (HPA) axis recovery. 

Within 48 hours of corticosteroid initiation, the patient exhibited marked improvement in mental status. She became alert, conversational, and physically interactive with staff. The previously observed tremor resolved, and her thought processes became organized and coherent. Although mild residual long-term memory deficits persisted, there was substantial overall improvement in cognitive function. Following recovery of baseline cognition, collateral history was obtained. The patient was re-interviewed and reported that her last alcohol intake occurred approximately two weeks prior, consisting of two glasses of wine at a social event. She recalled speaking with her brother five days before admission and confirmed the information provided by the collateral source. She confirmed that she lives with a roommate.

She reported experiencing intermittent palpitations and mental clouding during the week preceding admission. She did not recall events from the 36 hours prior to hospitalization or the circumstances surrounding her admission. She was independent in activities of daily living, worked as a logistics coordinator, and had no prior history of psychosis or cognitive impairment. Her only psychiatric history was an anxiety disorder, for which she was self-administering alprazolam 0.25 mg at bedtime intermittently. She had no known prior neurologic disease. Age-appropriate health screenings before admission were reported as up to date, including a normal mammogram and Pap smear performed one year prior, and a normal screening colonoscopy five years prior. Routine preventive evaluations, including blood pressure monitoring, lipid panel assessment, and screening for HIV and hepatitis C, were also within normal limits. The CIWA protocol was discontinued, and lorazepam was stopped due to improvement in clinical symptoms following steroid therapy, which made alcohol or benzodiazepine withdrawal less likely.

Her rapid clinical response to corticosteroid therapy strongly supported the diagnosis of SREAT in the setting of an acute neuropsychiatric presentation.

**Figure 2:**
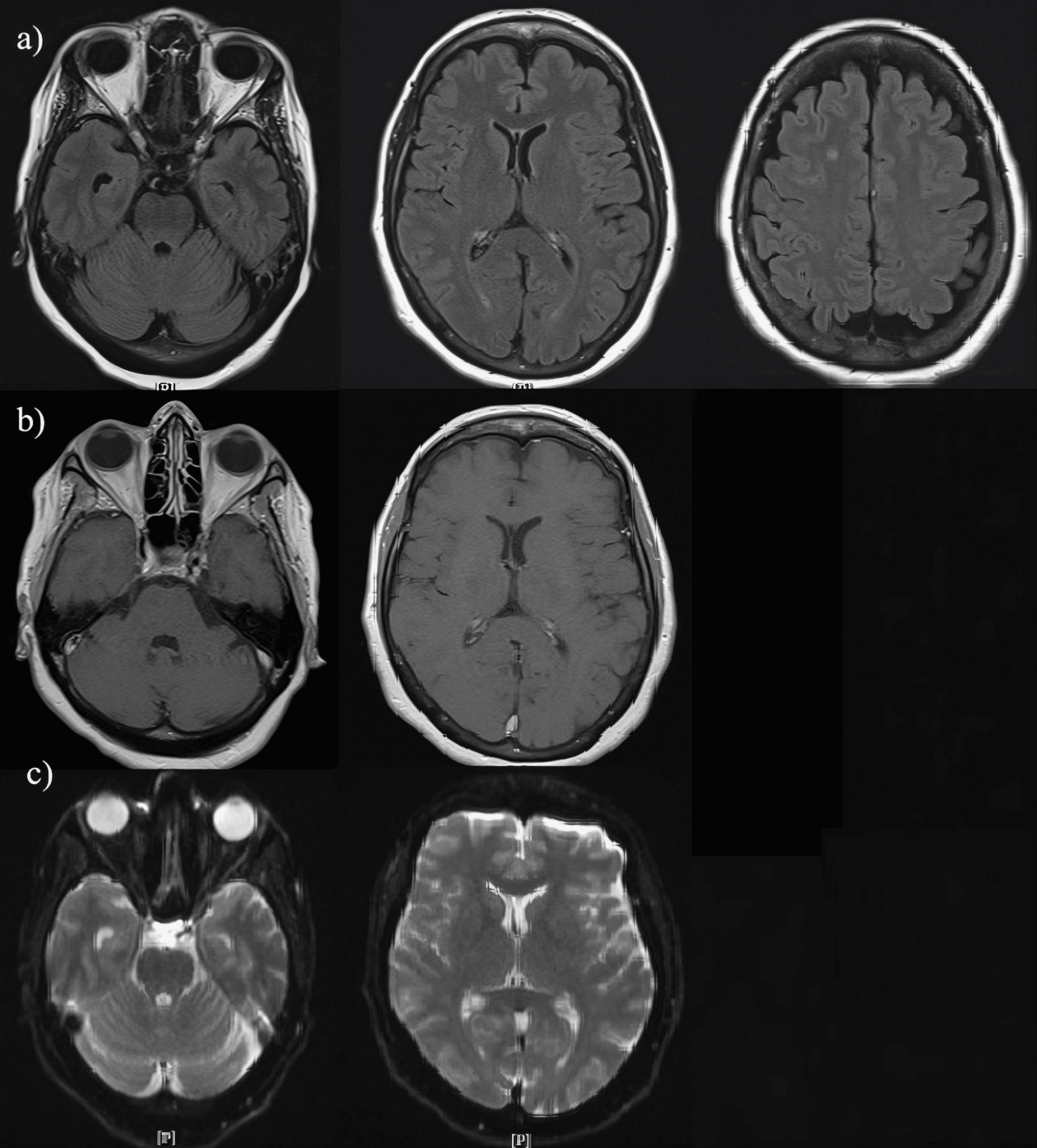
Multi-sequence brain MRI findings. (a) Axial T2-weighted fluid-attenuated inversion recovery (FLAIR) images showing no focal edema, demyelination, or signal abnormalities. Nonspecific scattered white matter changes consistent with mild chronic microangiopathic ischemia were noted. (b) T1-weighted post-contrast images demonstrating an absence of abnormal parenchymal or meningeal enhancement, suggesting an intact blood-brain barrier. (c) Diffusion-weighted imaging (DWI) confirming no evidence of acute ischemia or restricted diffusion.

Subsequent MRI of the brain with and without contrast showed no acute abnormalities aside from mild chronic microangiopathic changes (Figure 2). Electroencephalography was normal, and lumbar puncture revealed clear cerebrospinal fluid with normal glucose 85 mg/dl, protein 36 mg/dl, and WBC 1/mm³ (100% mononuclear cells), and a negative Gram stain; overall, not suggestive of bacterial meningitis or CNS inflammatory infiltration. Additionally, a comprehensive cerebrospinal fluid autoimmune and paraneoplastic encephalitis panel was obtained to exclude alternative autoimmune encephalopathies (Table 6). An infectious encephalitis workup was also reordered (Table 4).

**Table 6:** Specialized cerebrospinal fluid (CSF) and autoimmune encephalitis panel NMDAR: N-methyl-D-aspartate receptor; AMPAR: Alpha-amino-3-hydroxy-5-methyl-4-isoxazolepropionic acid receptor; GABA-B: Gamma-aminobutyric acid type B receptor; mGluR1: Metabotropic glutamate receptor 1; DPPX: Dipeptidyl-peptidase-like protein 6; LGI1: Leucine-rich glioma-inactivated protein 1; CASPR2: Contactin-associated protein-like 2; GAD65: Glutamic acid decarboxylase 65; IGLON5: IgLON family member 5; ANNA-1: Antineuronal nuclear antibody type 1; ANNA-2: Antineuronal nuclear antibody type 2; PCA-1: Purkinje cell antibody type 1; CRMP5: Collapsin response mediator protein 5; DNER: Delta and Notch-like epidermal growth factor-related receptor; ITPR1: Inositol 1,4,5-trisphosphate receptor type 1; AGNA-1: Anti-glial nuclear antibody type 1; GFAP: Glial fibrillary acidic protein; MOG IgG: Myelin oligodendrocyte glycoprotein immunoglobulin G; MBP: Myelin basic protein; CSF: Cerebrospinal fluid; IgG: Immunoglobulin G

Category	Test	Result	Reference Range
Synaptic/Autoimmune	NMDAR, AMPAR1, AMPAR2	Negative	Negative
	GABA-B receptor, mGluR1	Negative	Negative
	DPPX, LGI1, CASPR2	Negative	Negative
	GAD 65, IGLON5	Negative	Negative
Onconeural/Paraneoplastic	Hu (ANNA-1), Ri (ANNA-2), Yo (PCA-1)	Negative	Negative
	PCA-Tr (Tr), CRMP5 (CV2)	Negative	Negative
	Amphiphysin, Ma2/Ta, Zic4	Negative	Negative
	DNER, ITPR1, AGNA-1	Negative	Negative
Glial/Neuroimmunologic	GFAP, MOG IgG	Negative	Negative
	MBP (Myelin Basic Protein)	Negative	Negative
CSF Indices	Oligoclonal bands	None	None
	IgG Index	0.6	0.28–0.66
	Intrathecal synthesis	None	None
	Albumin ratio	Normal	<0.57 (age-adjusted)
	Blood-brain barrier	Intact	Intact

Endocrinologic reassessment demonstrated elevated TSH (7.08 μIU/mL) with thyroid autoimmunity, consistent with autoimmune thyroiditis with subclinical hypothyroidism. The endocrinology team recommended close outpatient hormonal follow-up at 2 weeks, followed by monthly evaluations for the first two months and subsequently at three-month intervals, with serial monitoring of thyroid function tests. This approach was advised, given the likelihood that the patient may require future levothyroxine 50 mcg oral replacement therapy once overt hypothyroidism develops. Additional pituitary markers revealed mild hyperprolactinemia (prolactin (PRL) 25.0 ng/mL), likely attributable to physiologic stress, medication exposure, or hypothalamic-pituitary axis dysregulation during acute encephalopathy, with follicle-stimulating hormone (FSH) 88.5 IU/L and luteinizing hormone (LH) 48.6 IU/L in the post-menopausal range. Insulin-like growth factor 1 (IGF-1) was low-normal (42 ng/mL) (Table 5). Confirmatory testing following the initial reactive HIV screening assay demonstrated no detectable viremia, with HIV-1 RNA PCR <20 copies/mL (undetected) and a repeat 4th-generation HIV antigen/antibody (Ag/Ab) assay returning NEGATIVE, confirming an originally false-positive screen. Given the concordance of both confirmatory tests, HIV infection was considered excluded in the acute setting. Repeat serologic testing in the outpatient setting was recommended to further confirm the false-positive nature of the initial result and to exclude an acute window-period infection. The immunologic profile showed CD4 635 cells/µL (25.4%), CD8 715 cells/µL (28.6%), and a CD4/CD8 ratio of 0.88 (Table 4). After 10 days of hospitalization, following completion of the five-day course of IV methylprednisolone and clinical stabilization with continued inpatient monitoring, the patient was discharged. Given her rapid clinical improvement following high-dose corticosteroid therapy, elevated thyroid autoantibodies, and exclusion of alternative infectious, metabolic, structural, and paraneoplastic causes, a clinical diagnosis of steroid-responsive encephalopathy associated with autoimmune thyroiditis (SREAT) was established. The patient was discharged on a prednisone taper, with multidisciplinary outpatient follow-up arranged, including neurology, endocrinology, and psychiatry follow-up, with regular monitoring of neurological status, thyroid function tests, and psychiatric symptoms. Reassessment in the outpatient setting by psychiatry and endocrinology at two weeks demonstrated continued psychiatric stability.

Psychiatry established a gradual taper of divalproex while continuing quetiapine 50 mg nightly; these medications were used for short-term symptomatic management of acute neuropsychiatric manifestations during recovery, rather than as treatment of a primary psychiatric disorder. Thyroid function tests remained consistent with subclinical hypothyroidism, and continued surveillance of thyroid parameters was recommended as previously outlined. This case underscores the diagnostic challenges of SREAT and highlights the importance of reconsidering autoimmune etiologies in unexplained neuropsychiatric presentations. A chronological summary of the patient’s clinical course, diagnostic evaluation, and response to treatment is presented below (Table 7).

**Table 7:** Clinical timeline illustrating the patient’s presentation, diagnostic workup, and rapid response to corticosteroid therapy in SREAT SREAT: Steroid-responsive encephalopathy associated with autoimmune thyroiditis; CSF: Cerebrospinal fluid; TPO: Thyroid peroxidase; CIWA: Clinical Institute Withdrawal Assessment for Alcohol

Clinical Phase	Timepoint	Clinical Details & Interventions	Status	Steroid Response
Symptom Onset	Pre-admission	Acute psychosis, behavioral disorganization, cognitive impairment; history of anxiety and severe alcohol use disorder	Baseline	
Admission	Day 0	Altered mental status, acute psychosis; differential includes alcohol withdrawal, metabolic disturbance, primary psychiatric disorder	Acute	
Early Management	Days 0–2	CIWA protocol, benzodiazepines (lorazepam), thiamine, IV fluids, empiric cefepime; no improvement	Acute	
Workup Phase	Days 1–3	Labs, MRI/CT, EEG, CSF analysis; largely non-diagnostic; reactive HIV screen, elevated B12	Diagnostic	
Turning Point	Day ~3	Elevated anti-TPO and anti-thyroglobulin antibodies; subclinical hypothyroidism; suspicion for SREAT	Critical	
Definitive Treatment	Days 3–4	Initiation of IV methylprednisolone 1 g daily	Recovery	Yes
Clinical Response	Within 48h	Rapid improvement, resolution of psychosis and behavioral symptoms	Recovery	Yes
Stabilization	Days 5–7	Completion of IV steroids, transition to prednisone taper	Recovery	Yes
Discharge	Final Day	Improved; discharged on prednisone taper; follow-up arranged	Recovery	Yes
Follow-up	Outpatient	Sustained improvement, no recurrence of psychosis	Recovery	Yes

## Discussion

Diagnostic complexity and the pitfalls of early psychiatric framing

Diagnosing steroid-responsive encephalopathy associated with autoimmune thyroiditis (SREAT) remains inherently challenging due to its protean neuropsychiatric manifestations, absence of disease-specific biomarkers, and reliance on exclusionary criteria. The heterogeneity of clinical presentations frequently leads to diagnostic delays, particularly when psychiatric symptoms predominate. In the present case, marked confusion, disorganized thought processes, agitation, and memory impairment significantly limited the reliability of history-taking and hindered formal psychiatric evaluation, complicating clinical reasoning and obscuring the underlying encephalopathic process.

Given the diagnostic uncertainty, a comprehensive evaluation was undertaken to systematically exclude common medical, neurologic, infectious, toxic, and structural causes of encephalopathy. Routine laboratory studies, including complete blood count and metabolic panels, revealed no clinically significant electrolyte, hepatic, renal, or hematologic abnormalities. Structural etiologies were initially prioritized and excluded based on a non-contrast head CT demonstrating no acute pathology. Toxic causes were considered less likely based on a negative urine toxicology screen; however, standard panels may not detect all potential substances, and exposure to agents not captured by routine screening could not be definitively excluded. Malignancy and systemic organ dysfunction were considered based on cross-sectional imaging, including CT of the abdomen and pelvis and renal and abdominal ultrasonography, all of which were unremarkable; the absence of imaging abnormalities reduces, but does not fully exclude, an underlying neoplastic or visceral process.

Infectious etiologies were considered unlikely based on an extensive, though not exhaustive, serologic and molecular workup for common viral, bacterial, and opportunistic pathogens, as detailed above; clinical judgment and epidemiologic context were central to this assessment. HIV-1 RNA PCR was undetectable, and repeat HIV antigen/antibody testing was negative, confirming that the initial reactive screen represented a false-positive result. Additional investigations excluded viral encephalitides, including HSV-1/2, CMV, EBV, VZV, SARS-CoV-2, syphilis serology, and hepatitis A, B, and C panels. Although such testing is typically performed prior to the initiation of empiric corticosteroid therapy, in this case, it was deferred due to the patient’s progressive neurological deterioration, limited cooperation with diagnostic procedures, and a high clinical suspicion for an underlying autoimmune process.

Despite the largely unrevealing workup, several alternative explanations remained plausible. The patient’s history of alcohol use disorder raised concern for alcohol related encephalopathy, including Wernicke-Korsakoff syndrome, which presents with mental status changes, ocular dysfunction, and gait abnormalities and may progress to irreversible memory impairment if untreated, as well as delirium tremens, the most severe manifestation of alcohol withdrawal characterized by marked confusion, disorientation, and autonomic hyperactivity [21,22]. Additional factors, such as mild metabolic acidosis and hypoglycemia associated with starvation ketosis, warranted consideration, as acute metabolic derangements can result in encephalopathy with significant decline in consciousness and impaired responsiveness [23]. The elevated vitamin B12 level raised concern for an occult neoplastic process, given the established association between elevated plasma vitamin B12 levels and an increased risk of solid tumors and hematologic malignancies [19].

In this case, evaluation included review of a complete blood count, which demonstrated no cytopenias, morphologic abnormalities, or leukocytosis suggestive of a primary hematologic process, as well as cross-sectional abdominal imaging, which was unremarkable. Dedicated hematologic evaluation, including peripheral blood smear, serum protein electrophoresis, or bone marrow assessment, was not performed during the inpatient course, which represents a recognized limitation of the diagnostic evaluation. A primary psychiatric disorder also remained within the differential diagnosis, as functional cognitive disorders can present with genuine and disabling cognitive symptoms that are not attributable to structural brain disease, although formal assessment was limited by impaired cognition [24]. Each of these elements plausibly contributed to the overall clinical picture, thereby increasing diagnostic uncertainty.

Empiric treatment strategies, including high-dose thiamine repletion, CIWA-guided benzodiazepine therapy, IV fluids, and psychotropic medications, were initiated. However, despite these interventions, the patient’s cognitive and behavioral status progressively deteriorated, characterized by worsening agitation, disorientation, and functional impairment.

Even after SREAT emerged as the leading diagnostic consideration, further investigations were pursued to exclude other autoimmune and inflammatory conditions. EEG, cerebrospinal fluid analysis, autoimmune and paraneoplastic encephalitis panels, and repeat infectious evaluations were unremarkable. This absence of supportive findings further underscores the diagnostic challenge posed by SREAT, in which ancillary studies are often nonspecific or normal.

This case highlights how overlapping clinical features may obscure autoimmune causes of encephalopathy and delay appropriate treatment with immunotherapy, most often high-dose corticosteroids. It also illustrates the risks associated with premature psychiatric attribution, particularly in patients with unreliable histories or severe behavioral disturbances, which may mask underlying neurologic disease. These findings emphasize the importance of iterative diagnostic reassessment and maintaining a broad differential diagnosis in treatment-refractory neuropsychiatric presentations.

Pathophysiology

The pathophysiology of SREAT remains incompletely understood and is likely multifactorial. Although anti-thyroid antibodies define the syndrome, they are thought to represent markers of immune dysregulation rather than direct mediators of central nervous system injury [13]. Current evidence suggests that the process may involve cytokine-mediated inflammation, T-cell activation, blood-brain barrier dysfunction, and possible microvascular mechanisms [4].

In this case, MRI demonstrated mild chronic microangiopathic ischemic changes, which may reflect vascular vulnerability. However, their direct role in symptom development remains uncertain. Blood-brain barrier dysfunction may allow systemic immune mediators to access the central nervous system even when CSF findings appear normal [4,25]. The nonspecificity of CSF, MRI, and EEG findings likely reflects a diffuse inflammatory process rather than focal structural lesions detectable by conventional imaging [4].

Role of thyroid autoimmunity

A critical teaching point illustrated by this case is that SREAT is independent of thyroid functional status. Patients may present as euthyroid, hypothyroid (subclinically in our patient), or hyperthyroid, and thyroid function may fluctuate throughout the disease course [4]. This reinforces that thyroid hormone abnormalities are not the primary drivers of encephalopathy and should not exclude SREAT from consideration [26].

Negative TSI and TRAb testing helped exclude Graves disease and supported autoimmune thyroiditis as the underlying process [27]. Levothyroxine therapy was suggested by the endocrinology team for possible future initiation if progression to overt hypothyroidism occurs, with close outpatient monitoring of thyroid function [28]. Anti-thyroid peroxidase (TPO) and anti-thyroglobulin antibodies were not remeasured, as antibody titers do not reliably correlate with disease activity or treatment response [5].

Therapeutic response

The patient’s rapid improvement within 48 hours of high-dose IV methylprednisolone strongly supported an immune-mediated process and served a therapeutic, rather than diagnostic, role. Although the term “steroid-responsive” is embedded in the nomenclature of SREAT, steroid responsiveness is not a formal diagnostic criterion and should not be considered a defining feature of the disorder. This terminology may be misleading, as only a subset (31.6%) of patients demonstrated a complete response to corticosteroids in studies that apply strict diagnostic criteria [8].

Earlier literature reported higher response rates (60-91%) likely due to methodological bias, as steroid responsiveness was often incorporated into the diagnostic definition, creating circular reasoning and artificially inflating efficacy estimates [7]. Despite this, clinical improvement may occur within days in acute cases or weeks in subacute presentations, though complete recovery may require prolonged immunosuppression [29].

Relapse is well documented, particularly when corticosteroids are withdrawn abruptly, underscoring the importance of gradual dose reduction and close monitoring [9]. Alternative immunotherapies, including IV immunoglobulin, plasma exchange, rituximab, and immunosuppressive agents such as azathioprine, mycophenolate mofetil, and methotrexate, have shown benefit in refractory or relapsing cases [9,30]. Management should be individualized, accounting for initial treatment response, relapse risk, and steroid tolerance. This is due to the absence of standardized guidelines regarding optimal steroid dosing, treatment duration, or tapering protocols [13].

Comparison with existing literature and case contributions

Misdiagnosis at presentation is common in SREAT, with most published reports initially attributing their cases to a viral encephalitis, prion disease, or degenerative dementia prior to recognition of autoimmune encephalopathy [8]. In contrast, this case was distinguished by the presence of multiple competing diagnostic possibilities, including alcohol-related encephalopathy, metabolic disturbances, an initial reactive HIV screening assay, elevated vitamin B12 levels suggesting possible malignancy, and a clinical presentation resembling primary psychosis. The coexistence of these confounding factors contributed to significant diagnostic complexity and delayed consideration of autoimmune etiology.

Reactive HIV screening results have not been widely reported in SREAT and may represent false positivity or immune-mediated serologic cross-reactivity, though causality cannot be established from a single case. The presence of this finding, alongside other plausible alternative explanations, further illustrates how overlapping clinical features can obscure recognition of autoimmune encephalopathy.

Psychiatric manifestations are well documented in SREAT, including mood disturbances, psychosis, and behavioral changes, but most reported cases allow meaningful psychiatric evaluation [7,8]. In contrast, this patient’s disorganization and agitation prevented reliable assessment and increased the risk of premature psychiatric attribution, confirming how early diagnostic framing may delay identification of underlying neurologic disease.

Although SREAT has been reported across a wide range of thyroid functional states, prior reports typically describe thyroid status as relatively stable at presentation [6]. In our patient, thyroid function tests demonstrated elevated TSH consistent with autoimmune thyroiditis, highlighting the variable and evolving endocrine findings that may accompany this condition. This observation reinforces that thyroid hormone abnormalities do not consistently correlate with the severity of encephalopathic manifestations, and their presence or absence should not exclude consideration of SREAT in similar clinical scenarios.

This case highlights the importance of maintaining diagnostic vigilance in patients with rapidly progressive neuropsychiatric symptoms, particularly when multiple plausible etiologies coexist. Anchoring on psychiatric or metabolic explanations may delay recognition of treatable autoimmune encephalopathies such as SREAT. The patient’s rapid response to corticosteroids, despite unrevealing conventional studies, underscores the need to consider immune-mediated causes even in diagnostically complex scenarios. Greater awareness of SREAT is critical, as timely diagnosis and treatment can lead to meaningful neurological recovery and prevent prolonged morbidity.

Future research directions

Despite increasing recognition of SREAT, critical knowledge gaps persist that prevent timely diagnosis and optimal management, as illustrated by this case. Prospective studies are urgently needed to develop and validate standardized diagnostic criteria that can reliably predict steroid responsiveness and distinguish SREAT from psychiatric and substance-related conditions, in order to reduce diagnostic delays and prevent inappropriate treatment [13]. Biomarker discovery studies should identify disease-specific markers beyond thyroid antibodies, which are found in approximately 8% of healthy controls, with promising candidates including intrathecal CD4+ T cell activation for predicting relapsing disease [8,13]. Mechanistic research employing advanced neuroimaging, CSF proteomics, and animal models is essential to elucidate the underlying pathophysiology, which would enable targeted rather than empirical immunotherapy [31].

Randomized controlled trials are needed to establish optimal corticosteroid dosing and duration, as no treatment guidelines currently exist despite steroids being first-line therapy [7]. Finally, comparative effectiveness trials evaluating steroid-sparing immunotherapies, including IV immunoglobulin and rituximab, are critical for the 16% of patients who relapse, which would reduce long-term corticosteroid morbidity while maintaining disease control [7]. 

## Conclusions

This case illustrates the diagnostic challenge of SREAT in the setting of competing metabolic, psychiatric, and substance-related explanations for acute neuropsychiatric decline. The patient’s presentation was confounded by multiple plausible alternative diagnoses, including severe alcohol use disorder, metabolic disturbances, an initially reactive HIV screening assay, and features suggestive of primary psychiatric illness, all of which reasonably guided early management. However, the absence of structural, infectious, metabolic, and paraneoplastic etiologies, combined with markedly elevated anti-thyroid antibody titers and a rapid, sustained response to corticosteroid therapy, ultimately supported the diagnosis of SREAT. As demonstrated by this case, SREAT may present with neuropsychiatric manifestations severe enough to preclude reliable clinical assessment, substantially elevating the risk of misclassification as a primary psychiatric disorder, particularly in the presence of competing diagnostic explanations.

Thyroid functional status does not reliably correlate with neurologic involvement, as demonstrated by this patient’s subclinical hypothyroidism, and conventional diagnostic modalities, including MRI, EEG, and cerebrospinal fluid analysis, may be nondiagnostic despite their essential role in excluding alternative causes. This case further highlights how overlapping comorbidities and serologic abnormalities can obscure recognition of autoimmune encephalopathy and delay immunotherapy, while the patient’s rapid improvement following high-dose corticosteroids suggests the potential importance of early identification and treatment for meaningful neurologic recovery. Increased clinician awareness is essential to minimize diagnostic delays, particularly in treatment-refractory neuropsychiatric presentations, and further research is warranted to refine diagnostic frameworks, clarify immunopathogenic mechanisms, and establish evidence-based therapeutic protocols to optimize long-term outcomes.
